# Supplementary material for: Anthropogenic aerosols mask increases in US rainfall by greenhouse gases
Source: Nat Commun. 2024 Feb 22;15:1318. doi: 10.1038/s41467-024-45504-8 (PMC10884021; doi:10.1038/s41467-024-45504-8)
Supplement: Supplementary file 1 — Supplementary Information [file 41467_2024_45504_MOESM1_ESM.pdf]

Supplementary Information for: Anthropogenic aerosols mask increases in US rainfall by greenhouse gases

Mark D. Risser[1*†], William D. Collins[2,3†], Michael F. Wehner[4], Travis A. O'Brien[5,1], Huanping Huang[6,1] and Paul A. Ullrich[7,8]

[1]Climate and Ecosystem Sciences Division, Lawrence Berkeley National Lab, Berkeley, CA, USA.
[2]Earth and Environmental Sciences Area, Lawrence Berkeley National Lab, Berkeley, CA, USA.
[3]Department of Earth and Planetary Science, University of California, Berkeley, CA, USA.
[4]Applied Mathematics and Computational Research Division, Lawrence Berkeley National Lab, Berkeley, CA, USA.
[5]Department of Earth and Atmospheric Sciences, Indiana University, Bloomington, IN, USA.
[6]Department of Geography and Anthropology, Louisiana State University, Baton Rouge, LA, USA.
[7]Program for Climate Model Diagnosis & Intercomparison,Lawrence Livermore National Laboratory, Livermore, CA, USA.
[8]Department of Land, Air, and Water Resources, University of California, Davis, CA, USA.

*Corresponding author(s). E-mail(s): mdrisser@lbl.gov;
†These authors contributed equally to this work.

Supplemental tables

Table 1 A summary of the various hypotheses examined in [1], along with conclusions ("Conclusion") and a confidence statement ("Confidence").

	**Hypothesis**	**Conclusion**[1]	**Confidence**[2]
H1	Global GHG forcing is consistent with $\approx 2\%/K$ (for mean rainfall) and $\approx 6\%/K$ (for extreme precipitation).	Yes	*Likely*
H2	All cross-correlation terms in the forced component of seasonal precipitation are negligible.	Yes[4]	*Very likely*
H3	Individual forcings are non-negligible for secular trends in precipitation.	Yes[4] and no	*Likely* (GHG, aer); *About as likely as not* (stratO3, LULCC); *Unlikely* (nat)[5]
H4a	SO_2 is the dominant aerosol species for changes in precipitation.	Yes	*Likely*
H4b	emiso2 correlates with changes in precipitation as well as wetso2 and iso2.[3]	Yes	*Very likely*
H4c	Regionalized time series of emiso2 yield the same results as using either local or CONUS-wide estimates.	Yes	*Virtually certain*
H5	All interaction terms between climate drivers and external forcing agents are negligible.	Yes[4]	*Likely*
H6	Compensating errors in GHG and aerosol forcing are negligible.	Yes[4]	*Likely*
H7	The signal-to-noise ratio of seasonal precipitation is a constant with respect to warming.	Yes	*Virtually certain*

[1]Except for hypothesis H1, which applies globally, all statements and conclusions are strictly limited to the continental United States for the period covering 1900 to 2020.

[2]The confidence labels reflect the categories defined by [2]. See Appendix E of [1] for more information on how each confidence statement is determined.

[3]Note: "emiso2" refers to SO_2 emissions; "wetso2" refers to deposition of SO_2 by rainfall; "iso2" refers to column integrated SO_2 mass [3].

[4]Indicates cases where we fail to reject the null hypothesis versus cases where results are conclusive.

[5]"GHG" is synonymous with GHG; "aer" denotes anthropogenic aerosols; "stratO3" denotes human-induced changes in stratospheric ozone; "LULCC" refers to human land-use and land-cover change; and "nat" denotes the combined effects of solar variability and volcanic eruptions, two primary natural climate drivers.

Table 2 Number of ensemble members for each Global Climate Model with sufficient data[1] for CMIP6 historical ("hist"), CMIP6 piControl or esm-piControl ("pi"), and CMIP6 1pctCO2 ("1pct") simulations [4].

**Model**	*hist*	*pi*	*1pct*	**Model**	*hist*	*pi*	*1pct*
ACCESS-CM2[2]	3	1	1	GFDL-CM4	0	1	0
ACCESS-ESM1-5[2]	30	2	1	GFDL-ESM4[2]	1	2	1
AWI-ESM-1-1-LR[3]	1	1	1	GISS-E2-1-G	2	0	0
BCC-CSM2-MR	3	0	0	GISS-E2-2-G	0	1	1
BCC-ESM1	3	0	0	HadGEM3-GC31-LL[2]	5	1	4
CAMS-CSM1-0	1	0	0	HadGEM3-GC31-MM[2]	4	1	1
CanESM5[2]	50	3	6	IITM-ESM[3]	1	1	1
CESM2[2]	9	2	1	INM-CM4-8[2]	1	1	1
CESM2-FV2	3	1	0	INM-CM5-0[2]	10	1	1
CESM2-WACCM[2]	3	1	1	IPSL-CM5A2-INCA[3]	1	1	1
CESM2-WACCM-FV2[2]	3	1	1	IPSL-CM6A-LR[2]	32	2	1
CMCC-CM2-HR4	1	0	0	IPSL-CM6A-LR-INCA	1	0	0
CMCC-CM2-SR5[2]	1	1	1	KACE-1-0-G[3]	1	1	1
CMCC-ESM2[2]	1	1	1	MIROC-ES2L[2]	29	2	1
CNRM-CM6-1[2]	29	1	1	MIROC6[2]	43	1	1
CNRM-CM6-1-HR[2]	1	1	1	MPI-ESM-1-2-HAM[3]	3	1	1
CNRM-ESM2-1[2]	10	2	10	MPI-ESM1-2-HR[2]	10	1	1
E3SM-1-0	0	1	1	MPI-ESM1-2-LR[2]	10	2	1
EC-Earth3[2]	11	1	1	MRI-ESM2-0[3]	6	3	2
EC-Earth3-AerChem[3]	2	1	1	NESM3	5	0	1
EC-Earth3-CC[2]	1	2	1	NorCPM1	30	0	0
EC-Earth3-Veg[4]	3	1	1	NorESM2-LM[2]	3	1	1
EC-Earth3-Veg-LR	3	1	0	NorESM2-MM	3	0	1
FGOALS-f3-L	3	0	3	SAM0-UNICON[2]	1	1	1
FGOALS-g3	5	0	2	TaiESM1	1	1	0
FIO-ESM-2-0	0	0	0	UKESM1-0-LL[2]	16	2	4

[1]Sufficiency means that the output from each ensemble member available from the Earth System Grid Federation (ESGF; [5]) includes the daily and monthly precipitation rates and the monthly variables needed to calculate the low-frequency drivers in main text Eq. 2.

[2]Models with at least one ensemble member from historical, pi-Control (with at least 500 years), and 1pctCO2 (25 total).

[3]Models whose pi-Control runs are less than 500 years.

[4]Models with errors in emiso2 (vertically-integrated total emission of SO_2) files.

Table 3 Number of years for each Global Climate Model with daily precipitation data for each PDRMIP experiment [6]. Here, "base" corresponds to present-day conditions and "sulx5" corresponds to present-day SO_4 (sulfate aerosol) concentrations multiplied by a factor of 5; each of these configurations has both a prognostic SST ("coupled") and fixed SST ("fsst") experiment.

**Model**	**base-fsst**	**sulx5-fsst**	**base-coupled**	**sulx5-coupled**
CanESM2	14	14	99	99
GISS-E2-R	49	14	99	99
HadGEM2	30	30	100	100
IPSL-CM5A	29	29	99	99
MIROC-SPRINTARS	14	14	99	99
NCAR-CESM1-CAM4	30	29	120	109
NCAR-CESM1-CAM5	24	24	132	132
NorESM1	20	20	100	100

Supplemental figures

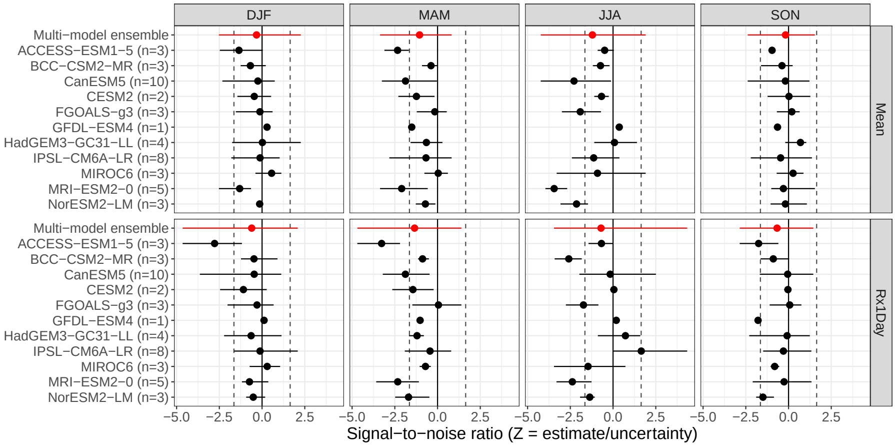

Fig. 1 Signal-to-noise ratio (SNR) estimates for the effect of anthropogenic aerosols on seasonal precipitation as simulated by individual ensemble members of the single-forcing hist-aer experiment [7]. Here we show the CONUS-average precipitation difference between 1851-1880 (representing pre-industrial conditions) and 1952-1981 (the 30 years in which SO_2 emissions were at their largest in CONUS). The plotted points show the ensemble average for each model (black) and the multimodel ensemble average (red), while the lines show the minimum and maximum SNR from each ensemble. The dashed vertical lines are at ±1.645, the SNR threshold corresponding to a significance level of $\alpha = 0.1$. Note that the multimodel ensemble average has very low SNR, failing to exceed the ±1.645 threshold in all seasons and for both mean and extreme precipitation, while individual ensemble members have statistically significant SNR that indicates both drying (< -1.645) and wetting (> 1.645) for seasonal precipitation at the CONUS scale.

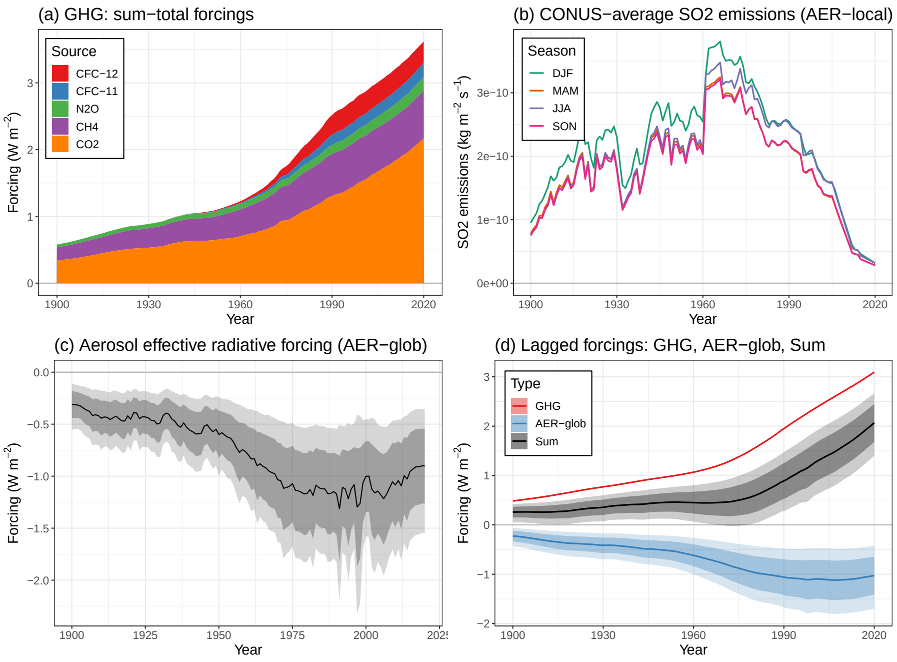

Fig. 2 Reconstructed best-estimate time series of external anthropogenic forcings used for the various analyses in this paper over 1900 to present day for well-mixed greenhouse gases (sum-total GHG forcings in W m^{-2}; panel a.) and CONUS-average SO_2 emissions, obtained from [8, 9], for each season (panel b.; March/April/May (MAM) and September/October/November (SON) are nearly overlapping).

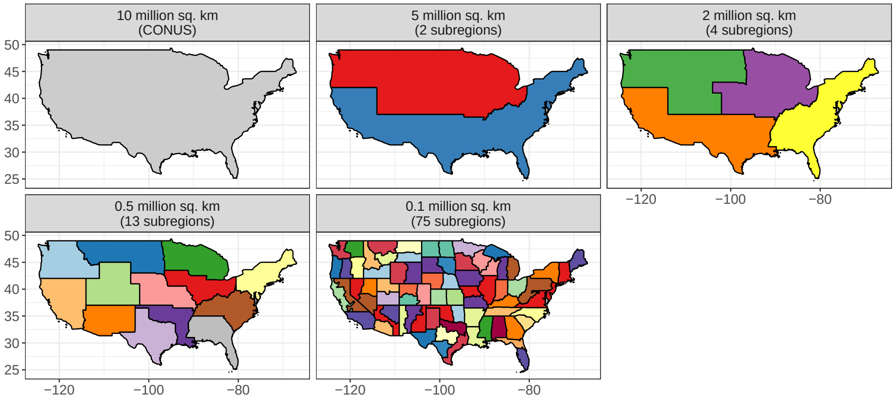

Fig. 3 Nested attribution regions, as defined in [10], that subsequently divided the CONUS into two, four, 13, and 75 subregions. The attribution regions correspond to spatial scales of $\approx 8\text{Mm}^2$ (all of CONUS), $\approx 4\text{Mm}^2$ (two subregions), $\approx 2\text{Mm}^2$ (four subregions), $\approx 0.5\text{Mm}^2$ (13 subregions), and $\approx 0.1\text{Mm}^2$ (75 subregions), where $1\text{Mm}^2 = 1$ million km^2; the grid boxes are $\approx 600\text{km}^2$.

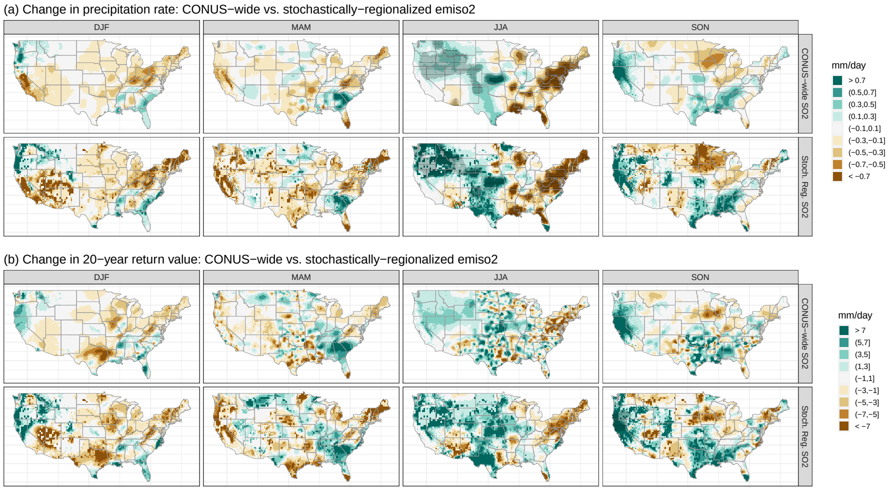

Fig. 4 Comparison of the estimated effect of SO_2 emissions on in-situ rainfall measurements when using a global CONUS-wide emissions time series (top row of panel a. and b.) versus the stochastically-regionalized emissions (bottom row of panel a. and b.). Note that the bottom row of each panel are as in Figure 2 in the main text. Hatching indicates where the changes are statistically significant, where a − (+) indicates moderate (strong) significance.

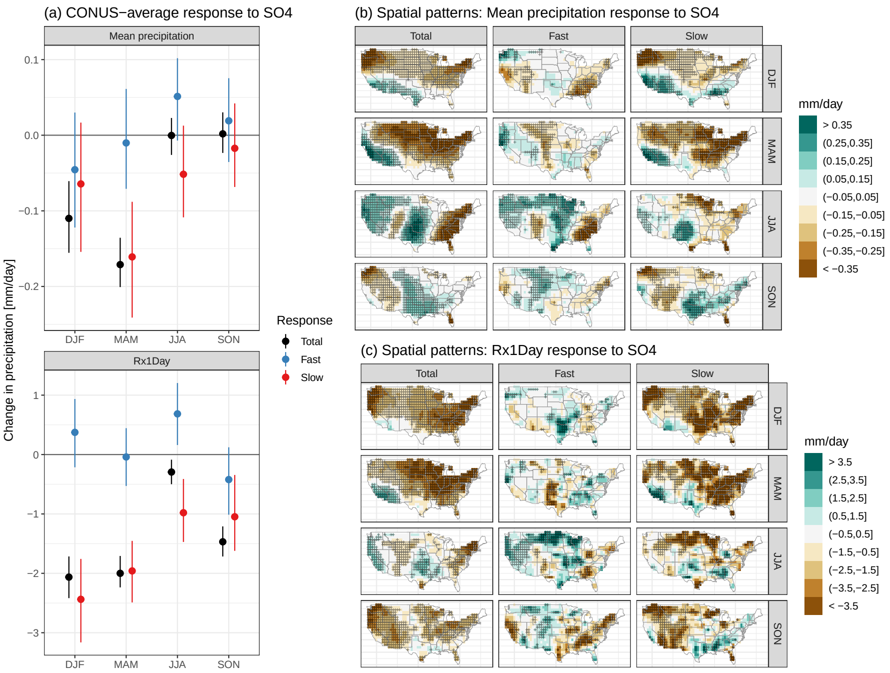

Fig. 5 Multi-model mean estimates of the fast, slow, and total precipitation response to sulfate aerosols, derived from experiments in the Precipitation Driver and Response Model Intercomparison Project [PDRMIP; 6]. Analogous to the results in Figures 3 and 4 in [11] but focusing on seasonal mean and extreme precipitation, we show CONUS-wide averages (panel a) as well as spatial patterns of response (panel b). Hatching indicates that the 90% bootstrap confidence interval does not include zero. Calculations are described in "Fast versus slow precipitation response to aerosols" in Methods; the GCMs used are listed in Supplemental Table 3.

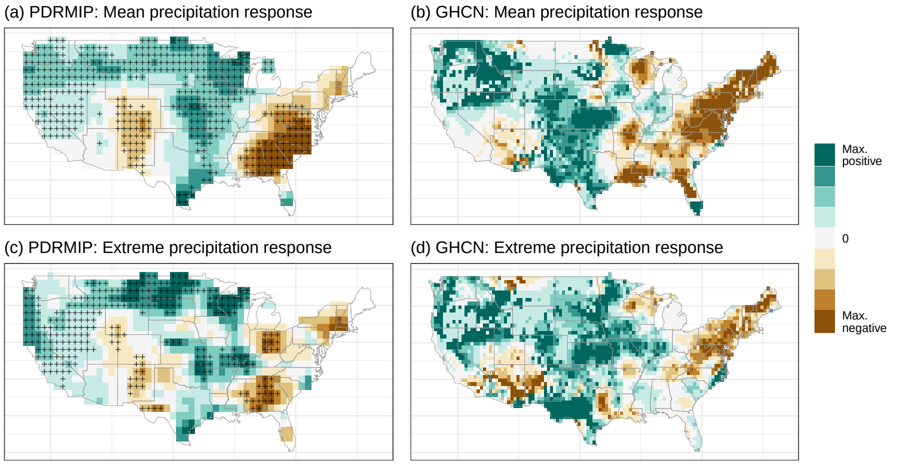

Fig. 6 Comparison of the summertime fast precipitation response to aerosols for our GHCN station-based analysis versus corresponding patterns from PDRMIP GCM simulations (showing the multi-model mean from eight GCMs). Thumbnail maps taken from Figure 2 in the main text and Supplemental Figure 5; note that the color bar limits in panels (a) vs. (b) and (c) vs. (d) are different due to the differing magnitude of SO_4 loadings in the simulations versus observations. (Note: hatching has different meanings in the GHCN vs. PDRMIP plots.)

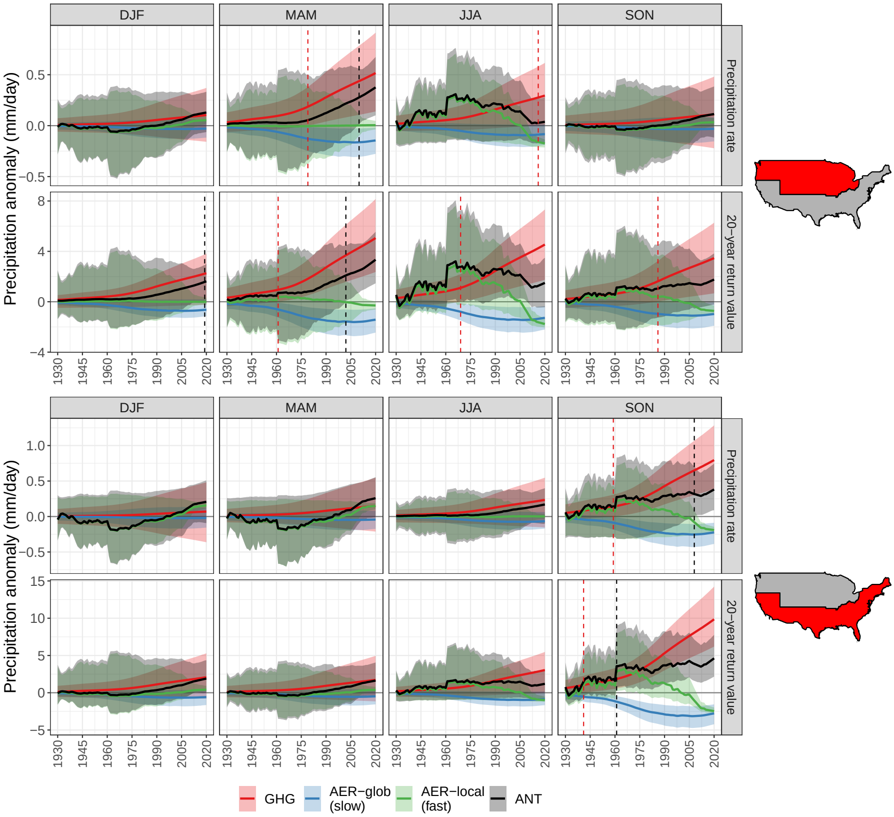

Fig. 7 Regionally-averaged time series of seasonal precipitation anomalies from the pre-industrial (1900) climate of mean and extreme precipitation for the two $\approx 4\text{Mm}^2$ subregions shown in the rightmost column. Each panel shows the isolated effect of anthropogenic forcing agents on seasonal precipitation (GHG, solid red line; AER-glob, the slow precipitation response to aerosols, solid blue line; AER-local, the fast precipitation response to aerosols, solid green line) as well as the combined anthropogenic response (ANT; solid black line) with a 90% bootstrap confidence band. Dashed vertical lines denote the year of emergence for isolated GHG signal (red) and combined ANT response (black), where emergence is defined as the first year in which the 90% confidence band departs from zero.

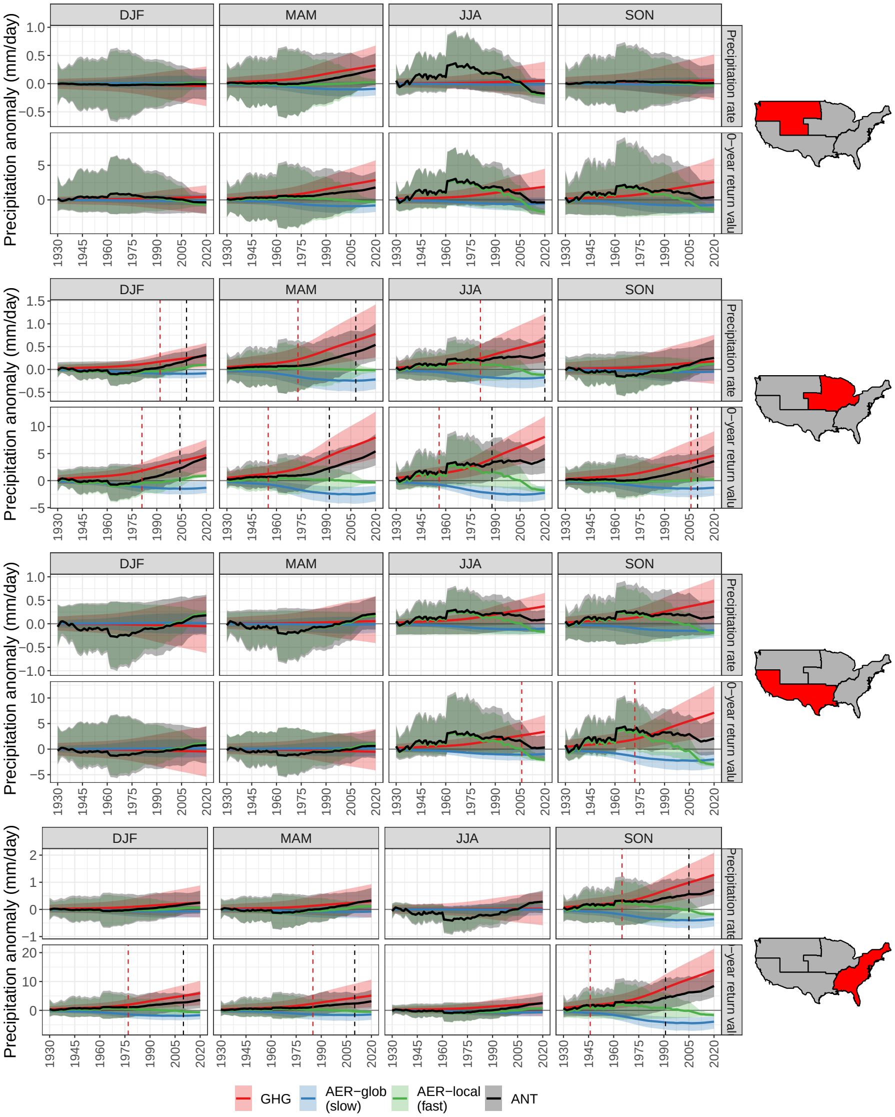

Fig. 8 Regionally-averaged time series of seasonal precipitation anomalies from the pre-industrial (1900) climate of mean and extreme precipitation for the four $\approx 2\text{Mm}^2$ subregions shown in the rightmost column. Each panel shows the isolated effect of anthropogenic forcing agents on seasonal precipitation (GHG, solid red line; AER-glob, the slow precipitation response to aerosols, solid blue line; AER-local, the fast precipitation response to aerosols, solid green line) as well as the combined anthropogenic response (ANT; solid black line) with a 90% bootstrap confidence band. Dashed vertical lines denote the year of emergence for isolated GHG signal (red) and combined ANT response (black), where emergence is defined as the first year in which the 90% confidence band departs from zero.

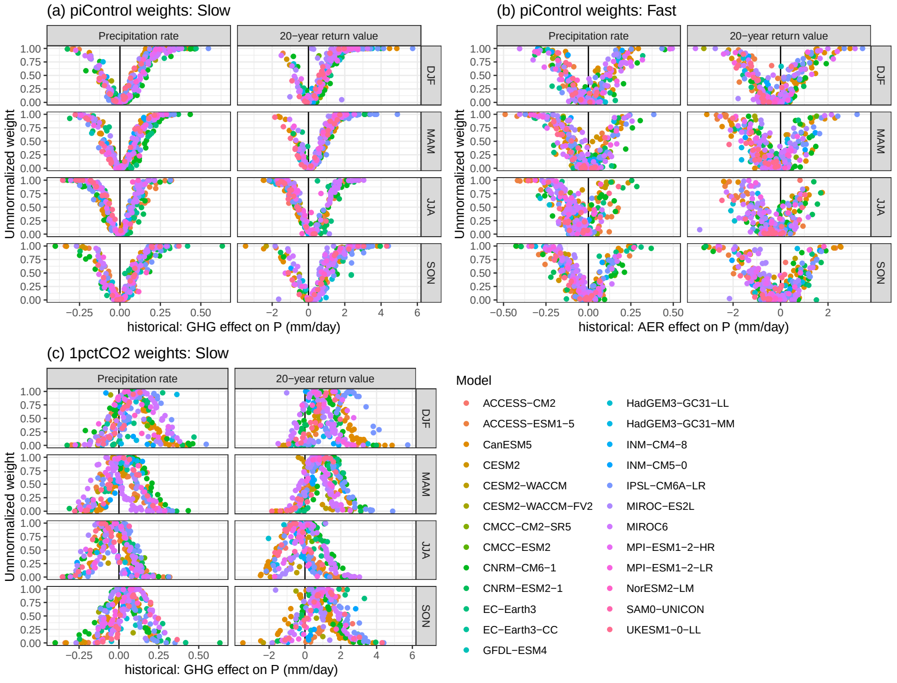

Fig. 9 Individual ensemble weights $w_{PI,\mathrm{GHG}}(j,m)$ (Eq. 13 in the main text; panel a.), $w_{PI,\mathrm{AER}}(j,m)$ (Eq. 13 in the main text; panel b.), and $w_{1\%}(j,m)$ (Eq. 15 in the main text; panel c.).

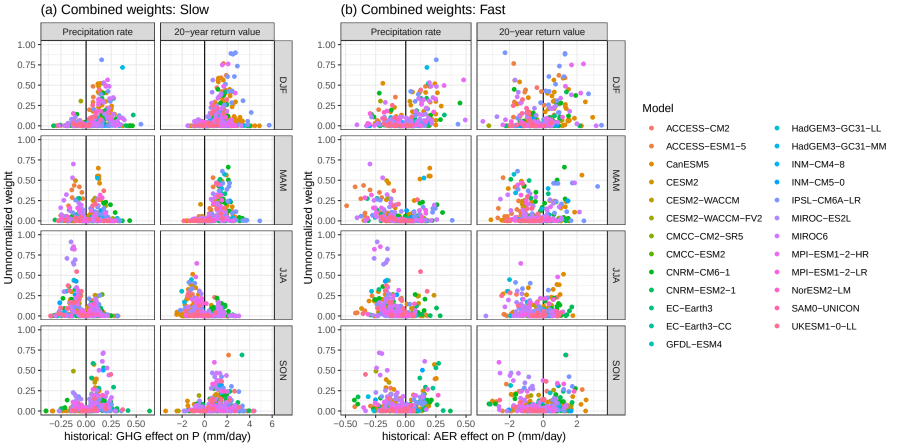

Fig. 10 Combined weights $w(j,m)$ (Eq. 17 in the main text), plotted versus estimates of the GHG effect on precipitation (panel a.) and the SO_2 effect on precipitation (panel b.).

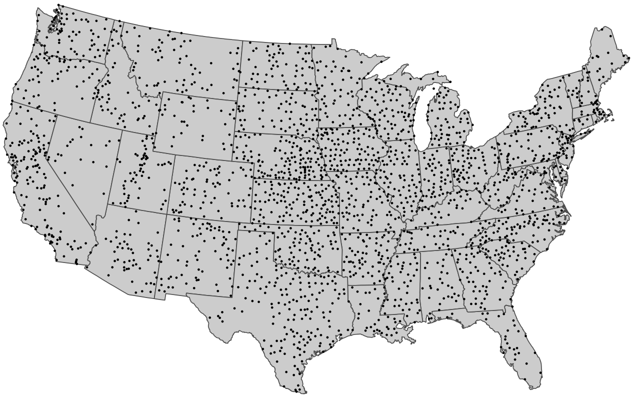

Fig. 11 The spatial distribution of the $n = 2480$ GHCN stations with a minimum of 66.7% of existent, quality-controlled daily precipitation measurements during the period spanning December 1899 to November 2020.

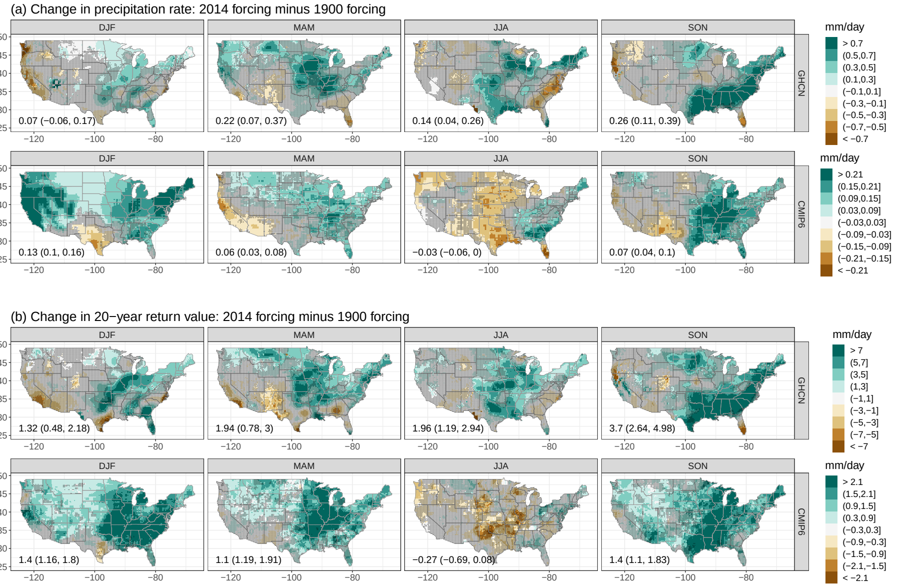

Fig. 12 Sum-total forced changes to seasonal mean (panel a.) and extreme (panel b.) precipitation for 2014 versus 1900 forcing levels (GHGs and SO_2 emissions) for the GHCN analysis (top row in each panel) and the weighted CMIP6-historical multimodel mean (bottom row in each panel). Each panel includes the area-weighted CONUS-mean change with uncertainty (best estimate and lower/upper 90% confidence bounds). Stippling indicates where the grid-box 90% confidence intervals include zero, i.e., the changes are indistinguishable from zero. Note that the color bar limits are different for the GHCN vs. CMIP6 maps.

References

[1] Risser, M.D., Collins, W.D., Wehner, M.F., O'Brien, T.A., Paciorek, C.J., O'Brien, J.P., Patricola, C.M., Huang, H., Ullrich, P.A., Loring, B.: A framework for detection and attribution of regional precipitation change: Application to the United States historical record. Climate Dynamics (2022). https://doi.org/10.1007/s00382-022-06321-1

[2] Mastrandrea, M.D., Field, C.B., Stocker, T.F., Edenhofer, O., Ebi, K.L., Frame, D.J., Held, H., Kriegler, E., Mach, K.J., Matschoss, P.R., et al.: Guidance note for lead authors of the IPCC Fifth Assessment Report on consistent treatment of uncertainties. Intergovernmental Panel on Climate Change. https://www.ipcc.ch/site/assets/uploads/2017/08/AR5_Uncertainty_Guidance_Note.pdf, verified 21. Jun. 2021 (2010)

[3] Centre for Environmental Data Analysis (CEDA): CMIP6 Data Request. http://clipc-services.ceda.ac.uk/dreq/mipVars.html. Accessed: 2021-07-21 (2016)

[4] Eyring, V., Bony, S., Meehl, G.A., Senior, C.A., Stevens, B., Stouffer, R.J., Taylor, K.E.: Overview of the Coupled Model Intercomparison Project Phase 6 (CMIP6) experimental design and organization. Geoscientific Model Development **9**(5), 1937–1958 (2016). https://doi.org/10.5194/gmd-9-1937-2016

[5] Petrie, R., Denvil, S., Ames, S., Levavasseur, G., Fiore, S., Allen, C., Antonio, F., Berger, K., Bretonnière, P.-A., Cinquini, L., Dart, E., Dwarakanath, P., Druken, K., Evans, B., Franchistéguy, L., Gardoll, S., Gerbier, E., Greenslade, M., Hassell, D., Iwi, A., Juckes, M., Kindermann, S., Lacinski, L., Mirto, M., Nasser, A.B., Nassisi, P., Nienhouse, E., Nikonov, S., Nuzzo, A., Richards, C., Ridzwan, S., Rixen, M., Serradell, K., Snow, K., Stephens, A., Stockhause, M., Vahlenkamp, H., Wagner, R.: Coordinating an operational data distribution network for CMIP6 data. Geoscientific Model Development **14**(1), 629–644 (2021). https://doi.org/10.5194/gmd-14-629-2021

[6] Myhre, G., Forster, P.M., Samset, B.H., Hodnebrog, Ø., Sillmann, J., Aalbergsjø, S.G., Andrews, T., Boucher, O., Faluvegi, G., Flaschner, D., Iversen, T., Kasoar, M., Kharin, V., Kirkevåg, A., Lamarque, J.-F., Olivié, D., Richardson, T.B., Shindell, D., Shine, K.P., Stjern, C.W., Takemura, T., Voulgarakis, A., Zwiers, F.: PDRMIP: A precipitation driver and response model intercomparison project—protocol and preliminary results. Bulletin of the American Meteorological Society **98**(6), 1185–1198 (2017). https://doi.org/10.1175/BAMS-D-16-0019.1

[7] Gillett, N.P., Shiogama, H., Funke, B., Hegerl, G., Knutti, R., Matthes, K., Santer, B.D., Stone, D., Tebaldi, C.: The detection and attribution

model intercomparison project (DAMIP v1. 0) contribution to CMIP6. Geoscientific Model Development **9**(10), 3685–3697 (2016). https://doi.org/10.5194/gmd-9-3685-2016

[8] Hoesly, R.M., Smith, S.J., Feng, L., Klimont, Z., Janssens-Maenhout, G., Pitkanen, T., Seibert, J.J., Vu, L., Andres, R.J., Bolt, R.M., Bond, T.C., Dawidowski, L., Kholod, N., Kurokawa, J.-I., Li, M., Liu, L., Lu, Z., Moura, M.C.P., O'Rourke, P.R., Zhang, Q.: Historical (1750–2014) anthropogenic emissions of reactive gases and aerosols from the Community Emissions Data System (CEDS). Geoscientific Model Development **11**(1), 369–408 (2018). https://doi.org/10.5194/gmd-11-369-2018

[9] Gidden, M., Riahi, K., Smith, S., Fujimori, S., Luderer, G., Kriegler, E., van Vuuren, D., van den Berg, M., Feng, L., Klein, D., Calvin, K., Doelman, J., Frank, S., Fricko, O., Harmsen, M., Hasegawa, T., Havlik, P., Hilaire, J., Hoesly, R., Horing, J., Popp, A., Stehfest, E., Takahashi, K.: input4MIPs.CMIP6.ScenarioMIP.IAMC.IAMC-IMAGE-ssp119-1-1. Earth System Grid Federation (2018). https://doi.org/10.22033/ESGF/input4MIPs.2485

[10] Stone, D.A.: A hierarchical collection of political/economic regions for analysis of climate extremes. Climatic Change **155**(4), 639–656 (2019). https://doi.org/10.1007/s10584-019-02479-6

[11] Samset, B.H., Myhre, G., Forster, P.M., Hodnebrog, Ø., Andrews, T., Faluvegi, G., Fläschner, D., Kasoar, M., Kharin, V., Kirkevåg, A., Lamarque, J.-F., Olivié, D., Richardson, T., Shindell, D., Shine, K.P., Takemura, T., Voulgarakis, A.: Fast and slow precipitation responses to individual climate forcers: A PDRMIP multimodel study. Geophysical Research Letters **43**(6), 2782–2791 (2016). https://doi.org/10.1002/2016GL068064
